# Associations of Tobacco Use and Alcohol Drinking with Laryngeal and Hypopharyngeal Cancer Risks among Men in Karunagappally, Kerala, India -Karunagappally Cohort Study

**DOI:** 10.1371/journal.pone.0073716

**Published:** 2013-08-28

**Authors:** Padmavathy Amma Jayalekshmi, Athira Nandakumar, Suminori Akiba, Paleth Gangadharan, Chihaya Koriyama

**Affiliations:** 1 Regional Cancer Center, Trivandrum, Kerala, India; 2 Natural Background Radiation Cancer Registry, Karunagappally, Kerala, India; 3 Kagoshima University Graduate School of Medical and Dental Sciences, Kagoshima, Japan; 4 Amrita Institute of Medical Sciences, Cochin, Kerala, India; The University of Auckland, New Zealand

## Abstract

**Background:**

From among a cohort of 65,553 men aged 30–84 in Karunagappally Taluk, Kerala, India, 52 hypopharyngeal cancer cases and 85 laryngeal cancer cases were identified by the Karunagappally Cancer Registry during the period between 1990 and 2009.

**Methods:**

We conduct Poisson regression analysis of grouped data, taking into account age and education.

**Results:**

This study showed that the incidence rates of cancers of the hypopharynx and the larynx were strongly related to the number of bidis smoked a day (*P*<0.001 for both hypopharyngeal and laryngeal cancers) and duration of bidi smoking (P=0.009; P<0.001). Laryngeal cancer risk was significantly increased by bidi smoking (P<0.001), cigarette smoking (P=0.013) and regular alcohol use (P=0.005).

**Conclusion:**

The present study, the first cohort study to examine the association of hypopharyngeal and laryngeal cancer incidence rates with bidi smoking in South Asia, clearly showed dose–response relationships between those cancer risks and bidi smoking; larger amounts of bidi smoked a day and longer durations of bidi smoking increased the incidence rates of those cancers. Tobacco chewing was found not related to the risk of hypopharynx or larynx cancer.

## Introduction

The risks of hypopharyngeal and laryngeal cancers are strongly related to alcohol drinking and cigarette smoking [1]. The most popular tobacco smoked in southern India, including Kerala State and Tamil Nadu, is bidi, which is made of 0.15-0.25g of sun dried flaked tobacco rolled into a conical shape in a dried rectangular piece of Temburni leaf ( *Diospyros* *melanoxylon* ) and a thread securing the roll [2]. Tobacco chewing is practiced in different ways in India. Several studies have shown the significant risk for oral cancer among tobacco chewers. Recently, Gupta et al. reported the results of the cohort study of 99,570 individuals aged >= 35 years in Mumbai and showed that the mortality of oral and pharyngeal neoplasm’s was increased by bidi-smoking [3]. A multicentric case-control study from India also showed that bidi smoking was a strong risk factor for cancer of the hypopharynx (odds ratio = 6.8) and the supraglottis (odds ratio = 7.5). On the other hand, the association of those cancers with cigarette smoking was relatively weak. However, the effects of the two products were similar for glottic cancer: odds ratio was 5.3 for bidi smoking and was 5.7 for cigarette smoking. In addition, tobacco chewing was also related to the risk of this cancer among those who never smoked bidis or cigarettes [4].

The associations of alcohol drinking with hypopharyngeal and laryngeal cancers were well established in western countries but not in Asian societies [18]. However, recent case-control studies in India reported the relationship between laryngeal cancers and alcohol drinking [5,6]. A multicentric case-control study in India also showed that alcohol drinking increased the risk of hypopharyngeal cancer [4]. Further evaluation on those associations in a cohort study seems warranted.

To date, the risk factors of hypopharyngeal and laryngeal cancer have been evaluated mainly by case-control studies [1,5]. In the early 1990s, we established a cohort of virtually all the residents in Karunagappally to examine the risk of cancer in relation to natural radiation from thorium-containing monazite sand, lifestyles and other factors including socio economic status (SES) [6]. Natural radiation was not related to cancer incidence in the study population [7]. In order to understand the associations of the tobacco use and alcohol drinking with hypopharyngeal and laryngeal cancer risks, the present study analyzed the data obtained from the follow-up of a rural population in Karunagappally (Karunagappally cohort study). In the analysis, SES was adjusted because it is known to be a risk factor of cancer of the hypopharynx and larynx [8]. We focused on men since smoking is rare and the incidence rates of hypopharyngeal and laryngeal cancers were low among women in this rural population.

## Subjects and Methods

### Baseline survey

Karunagappally taluk (an administrative unit, corresponding to a county) is a coastal area consisting of 12 subunits (panchayats) of Kollam district in Kerala, India. According to 1991 census, this taluk had a population of 385,103 (191,149 males and 193,954 females) residing in an area of 192 Sq. km. In the late 1980s, we planned to establish a cohort of the entire residents in Karunagappally taluk. All the households (N=71674) in Karunagappally taluk were visited by 12 to 14 trained interviewers, starting from January 1, 1990 and ending on December 31, 1997. Using a 6-page standardized questionnaire, they collected information on socio-demographic factors, lifestyles, medical and family history, housing, residential history and so on. Socio-demographic factors included religion, family income in Rupees, education and occupation.

Regarding tobacco smoking interviewer inquired whether they never smoked bidi or cigarette, habitually smoked them in the past or habitually uses them currently. For those who answered to have smoked or currently smoking, they further enquired the age started smoking, the daily frequency of smoking of bidi and cigarette and the duration of the habit. For tobacco chewing, similar questions were asked. The use of alcohol was also inquired.

In addition, indoor and outdoor radiation levels were measured at the time of house visit by using portable scintillometers. However, our cohort study has shown that high-level natural radiation does not increase any cancer risk [7].

### Study Population

In total, this household survey collected personal information on 359,614 subjects in 71,674 households, which correspond to 93% of population and 94% of households in Karunagappally by the 1991 census. There were 69,943 men who were 30-84 years old at the time of interview. Those younger than 30 years of age were excluded from analysis since cancer risk was low in this range and the effect of smoking is not apparent until decades after starting smoking. Those aged 85 years or older were also excluded from the analysis since the elderly are less likely to seek medical care for malignancy, possibly resulting in lower completeness of cancer case ascertainment and lower accuracy of diagnosis. It was also difficult to collect accurate information on lifestyles on their early lives. Also excluded were the local Rare Earth factory workers, who might have been exposed to various occupational exposures (N=1,428). In addition, 136 subjects, who had died or had been diagnosed as cancer before base-line interview survey, were excluded from analysis. Further, those who died within 3 years of interview were also excluded from analysis since their lifestyles might have been affected by their health conditions. Thus, there were 65,553 subjects for statistical analysis.

### Cancer case ascertainment

Karunagappally Cancer Registry got an IRB approval for Regional Cancer center for our cohort study. Institutional Review Board of Kagoshima University Graduate School of Medical and Dental Sciences, Japan, also approved the present study.

We analyzed cancer incidence during the period between January 1, 1997 and December 31, 2009. Cancer cases among the cohort were ascertained by the cancer registry in Karunagappally, which was officially initiated as of January 1, 1990. The registry reports have been presented in “Cancer Incidence in Five Continents” vol. VII [9], vol. VIII [10] and vol. IX [11]. The major activities to identify cancer cases were i) monthly routine visits to the Regional Cancer Center (RCC) in Trivandrum, which is the comprehensive cancer centre in the state of Kerala, and more than half of cancer cases registered in Karunagappally cancer registry were those who sought medical treatment in RCC (unpublished data); ii) annual visits to Trivandrum Medical College Hospital in Trivandrum; iii) annual visits to major pathological laboratories in Karunagappally taluk and its neighboring areas, and in Trivandrum; iv) annual visits to all the hospitals and medical practitioners in Karunagappally taluk; v) 3-4 time visits to three primary health centers in the taluk, which have cancer screening facilities; vi) our clinics to provide monthly follow-up care for local cancer patients, which became popular because it provides cancer patients with palliative care and palliative care home service as well and; vii) our cancer screening camps conducted twice a year on average in all panchayats in the taluk. Our registry workers retrieved medical records and other relevant documents of cancer cases of Karunagappally residents diagnosed in the RCC and other medical facilities, and abstracted information on cancer cases diagnosed.

Death reports were obtained from the death registers kept in the vital statistics division of each panchayat. House visits of the deceased, to supplement information on cause of death, were started in 1997. The proportion of death certificate only cases, which are registry entries only on the basis of this death certificate information, in Karunagappally cancer registry was 14% during 1990-1994, 10% during 1993-1997 and 4.3% in males and 5.0% in females during 1998-2002 [9–11].

The extent of migration among cohort members was assessed by conducting a door-to-door survey of all the households in the 6 panchayats (Chavara, Neendakara, Panmana, Alappad, Oachira and Thevalakkara) and in the remaining 6 panchayats in 2001 and 2003, respectively. The survey findings were linked to incident cases through name, address, age, house number etc. This survey showed that migration was negligible. Movement within a panchayat was 9.5% while migration to outside the taluk was 6% in the 13-year study period. Only 0.7% of cohort members were lost to follow-up. The majority of migration took place for job opportunities in Gulf countries.

According to the cancer incidence data reported by Karunagappally cancer registry, incidence rates (per 100,000) of cancers of the hypopharynx and larynx for men in Karunagappally during the period 1993-1997 were 1.9 and 2.9, respectively. Corresponding rates for women were 0.3 and 0.0, respectively [10]. Kerala is among the states with lowest incidence rates of those cancers. The age-standardized rates (ASR) of hypopharyngeal and laryngeal cancers among men in Karunagappally were 2.1 and 3.5, respectively. Corresponding rates in women were 0.3 and 0.0, respectively. Those rates are several fold lower than those in the states with high incidence rate of those cancers [10].

### Statistical analysis

Statistical analysis was based on the data in cross-classifications by attained age (5-year category), and other covariates. Relative risk (RR) and 95% confidence intervals (95%CI) were obtained from Poisson regression analysis of grouped survival data [12]; using the DATAB and AMFIT procedures of Epicure program.

In the analysis examining the association of cancer risk with bidi smoking, which has the three categories (never, former and current), the following model was used to estimate the RRs of former bidi smoker (represented by S_2_) and current-smokers (represented by S_3_):

H_0_(attained age, income) exp (β_2_S_2_+ β_3_S_3_),

where H_0_ represents the baseline, or background, cancer incidence (e.g., incidence rate for never smokers) for cross-classified strata by attained age and socio-demographic variables.

Religion was divided into Hindu, Christian and Muslim. In the study area there is virtually no other religion. Education was divided on the basis of schooling pattern in the study area. Occupation was broadly categorized into five groups. The grouping of monthly income was the one used in our questionnaire (which was decided based on preliminary surveys). Attained age at the time of the mid-point of one-year interval during the observational period (1990-2005) was calculated for each cohort members by the DATAB procedure of EPICURE program. The maximum likelihood estimates of β_2_ and β_3_, for example, are log relative risks (RRs) for the indicator variables S_2_ and S_3_, respectively, when compared to the reference category of S_1,_ adjusting for attained age and family income. Heterogeneity test was based on a global p-value for a set of indicator variables. Trend test was conducted by assigning the mean duration of, e.g., duration of tobacco chewing, in each category of duration.

The entry into the cohort was January 1^st^, 1990 or the date of interview, which was started on January 1, 1990 and ended on December 31, 1997. A member of the cohort was considered to be censored when he/she was diagnosed as cancers other than hypopharyngeal or laryngeal cancer or died of causes other than hypopharyngeal or laryngeal cancer. Thus, the end of follow-up was the date of diagnosis for cancer cases, the date of death for those deceased, the end of follow-up (December 31, 2005) or the date attaining age of 85. In person-year calculation, we ignored the migration of cohort members since information on migration was available only for a part of our observation period (1997-2005). The use of available information on migration in person-year calculation caused only small changes in relative risk estimates.

## Results

The present study examined 65,553 men aged 30-84 years old. By the end of 2009, 52 cases of hypopharyngeal cancer (ICD9: 148) and 85 cases of laryngeal cancer (ICD9: 161) were identified. Table 1 shows the sociodemographic features of study subjects. In the analysis adjusting for attained age none of SES related factors was significantly related to cancer risk. Hypopharyngeal cancer risk tended to be higher among those with lower level of income, and laryngeal cancer risk tended to be increased among those with lower education levels. However, neither of them was a statistically significant association.

**Table 1:** Sociodemographic features of study subjects (men).

	N	PYs*	Hypopharyngeal cancer case	RR	95%CI	Laryngeal cancer case	RR	95%CI
**Total**	65553	900720	52			85		
**Religion**				**P>0.5**			**P>0.5**	
Hindu	47689	658303	40	1	reference	65	1	reference
Moslem	11841	160475	9	1.0	0.5-2.0	11	0.7	0.4-1.4
Christian	6023	81942	3	0.6	0.2-1.8	9	1.1	0.5-2.1
Family income(Rs**)				**P=0.078**			**P>0.5**	
<500	4367	66066	3	0.5	0.2-1.7	7	1.0	0.4-2.3
501-1200	19460	281461	25	1	reference	30	1	reference
1201-2500	24794	332258	13	0.4	0.2-0.8	25	0.7	0.4-1.1
2501-3500	10839	141566	8	0.5	0.2-1.2	16	0.9	0.5-1.7
3500+	6093	79370	3	0.4	0.1-1.2	7	0.7	0.3-1.6
**Education**				**P=0.284**			**P=0.090**	
illiterate	4143	52337	3	0.5	0.1-1.5	4	0.4	0.1-1.2
primary school	16917	221434	24	1	reference	34	1	reference
middle school	17310	238947	11	0.6	0.3-1.2	28	1.0	0.6-1.7
high school	20775	298699	10	0.6	0.3-1.3	17	0.8	0.4-1.4
college	5703	79877	2	0.4	0.1-1.9	2	0.3	0.1-1.3
unknown	705	9427	2	2.3	0.5-9.7	0		
**Occupation**				**P=0.496**			**P=0.353**	
fishermen &farmers	12371	169365	9	1	reference	22	1	reference
unemployed	15070	200454	12	1.2	0.5-2.8	14	0.6	0.3-1.1
students	868	11802	0			1	0.6	0.1-4.5
skilled workers	33358	465066	29	1.5	0.7-3.2	41	0.9	0.5-1.4
others	3886	54033	2	0.9	0.2-4.0	7	1.2	0.5-2.8

* Monthly income. Person-years

** One rupee is 2-3 cents in US dollars.

Relative risk was obtained from the following model:

H = H_s_ exp (B_i_X_i_), where background hazard, Hs, was stratified by attained age, income and education; and X_i_ are categorical variables of sociodemographic factors.

Table 2 summarizes the results of risk analysis with respect to tobacco use and alcohol drinking. Those analyses were stratified on attained age, income and education. Bidi smoking was significantly related to the risks of hypopharyngeal cancer (P<0.001) and laryngeal cancer (P<0.001). Laryngeal cancer risk was significantly related to cigarettes smoking (P=0.013) and regular alcohol use (P=0.005). Tobacco chewing was not related to hypopharyngeal or laryngeal cancer in this series.

**Table 2:** Hypopharyngeal and laryngeal cancer risk in relation to tobacco use and alcohol drinking among men.

			Hypopharyngeal cancer			Laryngeal cancer		
	N	PYs*	Case	RR	95%CI	Case	RR	95%CI
**Bidi smoking**				**P=0.001**			**P<0.001**	
never	31277	441290	8	1	reference	11	1	reference
former	5830	70584	5	1.9	0.6-6.0	9	2.5	1.0-6.3
current	25403	347383	34	4.0	1.8-9.0	60	5.5	2.8-10.8
unknown	3043	41464	5	5.8	1.8-18.5	5	5.1	1.7-14.9
**Cigarette smoking**				**P=0.124**			**P=0.013**	
never	29205	398841	21	1	reference	34	1	reference
former	5603	71488	3	0.6	0.2-2.0	5	0.6	0.2-1.5
current	27835	390298	26	1.6	0.9-2.8	44	1.7	1.1-2.7
unknown	2910	40093	2	1.2	0.3-5.3	2	0.9	0.2-3.6
**Tobacco chewing**				**P>0.5**			**P>0.5**	
never	42190	582656	34	1	reference	51	1	reference
former	4383	54094	3	0.5	0.2-1.8	10	1.2	0.6-2.4
current	18568	258317	15	0.9	0.5-1.7	23	0.9	0.6-1.5
unknown	412	5653	0			1	2.5	0.3-18.1
**Alcohol drinking**				**P>0.5**			**P=0.005**	
never	33296	454553	23	1	reference	27	1	reference
former	7857	98248	9	1.2	0.6-2.6	19	2.0	1.1-3.7
current	24399	347905	20	1.3	0.7-2.4	39	2.1	1.3-3.5
unknown	1	14	0			0		

* Person years

Relative risk was obtained from the following model:

H = H_s_ exp (B_i_X_i_), where background hazard, Hs, was stratified by attained age income and education; and X_i_ are categorical variables of tobacco use or alcohol drinking.

Categories for unknown were excluded when calculating p values.

Table 3 summarizes the results of risk analysis regarding the combined effects between bidi smoking, cigarette smoking, tobacco chewing and alcohol drinking. Tobacco chewing was not related to the risk of hypopharyngeal or laryngeal cancer even in this approach. The association of neither cancer risk with bidi smoking was magnified by cigarette smoking, tobacco chewing or alcohol drinking; Bidi smoking was related to the risk of hypopharyngeal and laryngeal cancers regardless of the status of cigarette smoking, tobacco chewing or alcohol drinking. On the other hand, cigarette smoking increased neither cancer risk among non-bidi-smokers or among bidi-smokers (Table 3). Alcohol drinking increased hypopharyngeal cancer risk among non-tobacco chewers but the increase was not statistically significant. Interestingly, the increase of laryngeal cancer risk by alcohol drinking appeared to be magnified by bidi smoking, cigarette smoking and tobacco chewing. However, none of those modifying effects was statistically significant.

**Table 3:** The combined effects of tobacco chewing, bidi/cigarette smoking, and alcohol drinking on incidence rates of hypopharyngeal and laryngeal cancers among men.

			Hypopharyngeal cancer			Laryngeal cancer		
		PYs	Case	RR	95%CI	case	RR	95%CI
**Cigarette smoking**	**Bidi smoking**							
never	never	267320	5	1	reference	7	1	reference
	current	99803	11	3.1	1.0-9.4	20	4.4	1.8-10.8
current	never	139294	3	1.4	0.3-5.8	4	1.3	0.4-4.4
	current	246681	23	4.1	1.5-11.0	40	5.4	2.4-12.3
**Tobacco chewing**	**Bidi smoking**							
never	never	321601	5	1	reference	8	1	reference
	current	199229	23	4.9	1.8-13.5	37	5.3	2.3-11.8
current	never	102489	2	1.1	0.2-5.5	3	1.0	0.3-3.9
	current	122623	9	3.6	1.2-11.4	16	4.2	1.7-10.3
**Alcohol drinking**	**Bidi smoking**							
never	never	279667	6	1	reference	8	1	reference
	current	129096	14	2.8	1.0-7.6	13	2.4	1.0-5.9
current	never	134013	1	0.4	0.1-3.6	2	0.6	0.1-2.8
	current	174429	14	2.8	1.0-7.5	33	5.7	2.6-12.8
**Tobacco chewing**	**Cigarette smoking**							
never	never	279202	16	1	reference	20	1	reference
	current	238526	16	1.3	0.6-2.6	29	2.0	1.1-3.6
current	never	100856	4	0.5	0.2-1.6	10	1.1	0.5-2.4
	current	127294	8	1.3	0.5-3.0	11	1.5	0.7-3.1
**Alcohol drinking**	**Cigarette smoking**							
never	never	276668	11	1	reference	16	1	reference
	current	135909	11	2.1	0.9-5.0	9	1.4	0.6-3.2
current	never	92346	6	1.5	0.5-4.0	11	2.0	1.2-4.4
	current	211995	10	1.5	0.6-3.6	26	2.9	1.5-5.5
**Alcohol drinking**	**Tobacco chewing**							
never	never	340933	14	1	reference	18	1	reference
	current	93024	7	1.6	0.6-4.0	6	1.1	0.4-2.7
current	never	192077	15	2.3	1.1-5.0	25	2.9	1.5-5.3
	current	135288	5	1.1	0.3-2.8	11	1.7	0.8-3.5

PYs: Person-years of observation

Relative risk was obtained from the following model:

H = H_s_ exp (B_i_X_i_), where background hazard, Hs, was stratified by attained age, income and education; and X_i_ are categorical variables created by combination of tobacco chewing and bidi smoking/alcohol drinking.

The results of further analyses on the association with bidi smoking are summarized in Table 4. Larger amounts of bidis smoked a day were related to the risks of hypopharyngeal (P<0.001) and laryngeal cancers (P<0.001). Longer duration of bidis smoked a day were related to the risks of hypopharyngeal (P=0.009) and laryngeal cancers (P<0.001).

**Table 4:** Bidi smoking and hypopharyngeal and laryngeal cancer risk among men.

		Hypopharyngeal cancer			Laryngeal cancer		
	PY’s	case	RR	95%CI	case	RR	95%CI
**Bidi smoked a day**			P for trend** < 0.001			P for trend<0.001	
Never	441290	8	1	reference	11	1	reference
Former	70584	5	1.9	0.6-6.1	9	2.6	1.1-6.5
1-4	40768	3	3.6	0.9-13.8	0		
5-14	131494	9	2.8	1.0-7.6	17	4.4	2.0-9.7
15-24	105359	10	3.8	1.5-10.1	25	7.6	3.6-16.1
25+	67303	12	7.0	2.7-18.2	18	8.5	3.8-19.0
unknown	43924	5	5.6	1.8-17.7	5	5.0	1.7-14.6
**Duration of bidi smoking**			P for trend* = 0.009			P for trend<0.001	
never	441290	8	1	reference	11	1	reference
1-14	141330	3	1.8	0.4-7.1	6	3.1	1.1-9.0
15-29	123435	8	3.0	1.1-8.1	17	5.2	2.4-11.5
30-44	84545	20	6.4	2.7-15.0	23	5.1	2.4-10.8
45+	68393	8	2.0	0.7-5.9	23	5.2	2.4-11.7
unknown	41728	5	5.5	1.7-17.4	5	5.0	1.7-15
**Age at starting of bidi smoking**			P for trend*** > 0.5			P for trend*** = 0.234	
<18	441290	8	3.5	1.1-11.0	11	6.3	2.7-14.7
18-	64800	6	4.2	1.8-10.2	13	5.6	2.7-11.6
23+	172714	18	3.3	1.3-8.6	31	4.1	1.8-9.0
never	109709	10	1	reference	16	1	reference
unknown	41624	5			5		

− former smokers were excluded from analysis

* PYs: Person-years of observation, ** Those in “unknown” category were excluded when calculating P for trend. Former smokers were also excluded when calculating P for trend for bidis smoked a day.

*** P for trend was calculated using only categories of <18, 18-, and 23+.

Relative risk was obtained from the following model: H = H_s_ exp (B_i_X_i_), where background hazard, Hs, was stratified by attained age (5 year category), income and education. X_i_ are categorical variables for bidi smoking.

The results of further analyses on the association with cigarette smoking are summarized in Table 5. The numbers of cigarettes smoked a day was related to an increased risk of laryngeal cancer (P=0.017). A similar association without a statistical significance was observed for hypopharyngeal cancer (P=0.153) as well. Neither the risk of hypopharyngeal cancer nor laryngeal cancer increased significantly with the daily amount of drinking or duration of drinking (data not shown).

**Table 5:** Cigarette smoking and tobacco chewing in relation to hypopharyngeal and laryngeal cancer risks among men.

			Hypopharyngeal cancer			Laryngeal cancer		
		PY’s	case	RR	95%CI	case	RR	95%CI
**Cigarette smoking**								
**Cigarettes smoked a day**				P for trend** =0.153			P for trend =0.017	
never		398841	21	1	reference	34	1	reference
former		71488	3	0.6	0.2-2.0	5	0.6	0.2-1.5
1-4		178945	13	1.4	0.7-2.9	23	1.6	0.9-2.8
5-14		178795	12	1.8	0.9-3.8	18	1.8	1.0-3.1
15-24		34545	1	0.9	0.1-6.7	2	1.1	0.3-4.4
25+		8780	1	2.9	0.4-21.5	2	3.9	0.9-16.3
unknown		29327	1	0.9	0.1-6.7	1	0.6	0.1-4.7
**Duration of cigarette smoking**				P for trend** =0.213			P for trend =0.103	
never		398841	21	1	reference	34	1	reference
1-19		212691	4	0.9	0.3-2.7	7	1.1	0.4-2.5
20-29		134583	6	1.1	0.4-2.4	12	1.3	0.7-2.6
30-49		72749	12	2.1	1.0-4.1	18	1.6	0.9-3.2
40+		41727	7	1.4	0.5-3.4	12	1.4	0.7-2.9
unknown		40129	2	1.1	0.3-5.0	2	0.8	0.2-3.5
**Tobacco chewing**								
**daily frequency**				P for trend** >0.5			P for trend >0.5	
never	585267		34	1	reference	51	1	reference
former	54795		3	0.5	0.2-1.8	10	1.2	0.6-2.4
1-4	165615		6	0.6	0.3-1.5	14	1.0	0.5-1.8
5-14	73579		6	1.1	0.4-2.5	9	1.1	0.5-2.2
15+	10553		2	2.2	0.5-9.1	0		
unknown	15249		1	1.4	0.2-10.7	1	1.0	0.1-7.0
**Duration**				P for trend** >0.5			P for trend >0.5	
never	585267		34	1	reference	51	1	reference
1-19	180756		6	0.7	0.3-1.6	15	1.1	0.6-2.0
20-29	61343		4	0.8	0.3-2.4	7	1.0	0.4-2.2
30-39	34131		5	1.2	0.5-3.2	5	0.8	0.3-1.9
40+	21623		2	0.6	0.1-2.5	5	1.0	0.4-2.5
unknown	21939		1	0.9	0.1-6.4	2	1.2	0.3-4.8

* PYs: Person-years of observation

** Those in “unknown” category were excluded when calculating P for trend. Former smokers were also excluded when calculating P for trend for cigarettes smoked a day.

Relative risk was obtained from the following model: H = H_s_ exp (B_i_X_i_), where background hazard, Hs, was stratified by attained age (5 year category), income and education. X_i_s are categorical variables for cigarette smoking/tobacco chewing.

## Discussion

The present cohort study has confirmed that bidi smoking increases the risk of hypopharyngeal and laryngeal cancer, as we observed in our previous study for lung cancer [13]. The larger numbers of bidis smoked a day and the longer duration of bidi smoking increased those cancer risks.

Cigarette smoking, the most common form of tobacco use, is known to be an important risk factor of hypopharyngeal and laryngeal cancers [5]. In the present study, cigarette smoking increased laryngeal cancer risk particularly among those who started smoking at ages younger than 18. Age at starting smoking seems to have more strong effects on laryngeal cancer risk among cigarette smokers when compared to bidi smokers.

The association with bidi smoking appeared to be slightly more evident for laryngeal cancer. However, the difference was not statistically significant. Comparison of hypopharyngeal and laryngeal cancer risk in relation to cigarette smoking was conducted by a few studies [14–18]. The IARC international study of cancers of the hypopharynx and larynx in Europe showed that the effect of cigarette smoking was similar to all sites. Another case-control study in France did not show any evident difference between cancers of hypopharynx and larynx in relation to cigarette smoking. A similar comparison in relation to bidi smoking conducted by the study of Sapkota et al. [4] did not show any evident difference between hypopharyngeal and laryngeal cancer risks, as was the case in the present study as shown in Table 3. In the case of hypopharyngeal cancer, those uses chewing tobacco 15 or more times a day had two-fold increase of risk although the increase was not statistically significant.

In the present study, tobacco chewing was not confirmed to be related to the risk of hypopharyngeal cancer or laryngeal cancer. Even if we restrict our analysis to those who never smoked bidis, so the RRs comparing current tobacco chewers and those who never chewed tobacco for cancers of the hypopharynx and larynx were 1.1 (95% CI=0.2-5.5) and 1.0 (95%CI= 0.3-3.9), respectively. For those who never smoked cigarettes, the corresponding RRs were 0.5 (95% CI=0.2-1.6) and 1.1 (95%CI= 0.5-2.4). However, in a multicentric case control study conducted in India, after restricting the analysis to never smokers, tobacco chewing showed a significantly increased risk of hypopharyngeal cancer [4]. The lack of risk may be due to facts that chewing tobacco in Kerala contains raw areca nuts and tobacco rather than cured ones and may be due to the duration of tobacco chewing [2].

Alcohol drinking was not evidently related to hypopharyngeal cancer risk in the present study, either, confirming the notion made by the review by P Boyle et al., which pointed out that alcohol drinking was an important risk factor in Western countries but not in Asian societies [17]. On the other hand, alcohol drinking was related to laryngeal cancer risk.

A study in France showed that education and occupation were related to hypopharyngeal and laryngeal cancer risk. In their study, the association with education was not significant once the effect of occupation was taken into account [18]. On the other hand, in the present study; illiterates had higher risks of hypopharyngeal and laryngeal cancers whereas occupation was related to neither cancer risk.

A disadvantage of a cohort study is the fact that the lifestyle of cohort members, examined at the start of its following-up, may change during follow-up. In the present study, no attempt was made to re-interview the cohort members. Generally speaking, however, the number of subjects who start smoking after age 30 is considered to be limited. Indeed, most of the bidi smokers examined in the present study took up smoking before age 30. On the other hand, it is difficult to estimate the number of subjects who quit smoking during follow-up. Because of such problems, the RRs for bidi smoking presented in the present study might have been underestimated.

In conclusion, the present study, the first cohort study to examine the association of hypopharyngeal and laryngeal cancer incidence rates with bidi smoking in South Asia, clearly showed dose–response relationships between those cancer risks and bidi smoking; larger amounts of bidi smoked a day and longer durations of bidi smoking increased the incidence rates of those cancers. In the present study, tobacco chewing was found not related to the risk of hypopharynx or larynx cancer. The lack of risk may be due to facts that chewing tobacco in Kerala contains raw areca nuts and tobacco rather than cured ones and may be due to the duration of tobacco chewing. Further studies seem warranted to examine to address this point.

WHO identified six evidence-based tobacco control measures that are the most effective in reducing tobacco use known as “MPOWER”. These measures are to monitor tobacco use and prevention policies, to protect people from tobacco smoke, to offer help to quit tobacco use, to warn people about the dangers of tobacco, to enforce bans on tobacco advertising, promotion and sponsorship, and to raise taxes on tobacco. The local and central governments of India should implement those measures to reduce health burden of tobacco use [19].

## References

[1] Sankaranarayanan R, Masuyer E, Swaminathan R, Ferlay J, Whelan S (1998) Head and neck cancer: a global perspective on epidemiology and prognosis. Anticancer Res 18: 4779-4786. PubMed: 9891557. 9891557

[2] Reddy KS, Gupta PC (2004) Report on tobacco control in India 5 New Delhi: Ministry of Health and Family Welfare, Government of India pp. 589-594.

[3] Gupta PC, Pednekar MS, Parkin DM, Sankaranarayanan R (2005) Tobacco associated mortality in Mumbai (Bombay) India. Results of the Bombay Cohort Study. Int J Epidemiol 34: 1395-1402. doi:10.1093/ije/dyi196. PubMed: 16249218. 16249218 10.1093/ije/dyi196

[4] Sapkota A, Gajalakshmi V, Jetly DH, Roychowdhury S, Dikshit RP et al. (2007) Smokeless tobacco and increased risk of hypopharyngeal and laryngeal cancers: a multicentric case-control study from India. Int J Cancer 121: 1793-1798. doi:10.1002/ijc.22832. PubMed: 17583577. 17583577 10.1002/ijc.22832

[5] IARC (2004) Tobacco smoke and involuntary smoking. IARC Monogr Eval Carcinog Risks Hum. 2004/08/03 ed. pp. 1-1438 PMC4781536 15285078

[6] Nair MK, Nambi KS, Amma NS, Gangadharan P, Jayalekshmi P et al. (1999) Population study in the high natural background radiation area in Kerala, India. Radiat Res 152: S145-S148. doi:10.2307/3580134. PubMed: 10564957. 10564957

[7] Nair RR, Rajan B, Akiba S, Jayalekshmi P, Nair MK et al. (2009) Background radiation and cancer incidence in Kerala, India-Karanagappally cohort study. Health Phys 96: 55-66. doi:10.1097/01.HP.0000327646.54923.11. PubMed: 19066487. 19066487 10.1097/01.HP.0000327646.54923.11

[8] Faggiano F, Partanen T, Kogevinas M, Boffetta P (1997) Socioeconomic differences in cancer incidence and mortality. IARC Sci Publ: 65-176. PubMed: 9353664. 9353664

[9] Nair MK, Mani KS (1997) Cancer incidence in Karunagapally 1990-1994.Cancer incidence in five continents. IARC Sci Publ. Volume VII: 1997 /01 /01 ed. pp. i-xxxiv, 1-1240 9505086

[10] Nair MK, Gangadharan P, Jayalakshmi P, Mani KS (2002) Cancer Incidence in Karunagapally 1993-1997, Kerala, India.Cancer incidence in five continents. IARC Sci Publ. Volume VIII: 2003/06/19 ed. pp. 240-241

[11] Jayalekshmi P, [!(surname)!] (2008) Cancer incidence in Karunagapally 1998-2002 Kerala, India.Cancer incidence in five continents. IARC Sci Publ. Volume IX: 1-837. 19388204

[12] Breslow NE, Day NE (1987) Statistical methods in cancer research. Volume II--The design and analysis of cohort studies. IARC Sci Publ: 1-406. 3329634

[13] Jayalekshmy PA, Akiba S, Nair MK, Gangadharan P, Rajan B et al. (2008) Bidi smoking and lung cancer incidence among males in Karunagappally cohort in Kerala, India. Int J Cancer 123: 1390-1397. doi:10.1002/ijc.23618. PubMed: 18623085. 18623085 10.1002/ijc.23618

[14] Tuyns AJ, Estève J, Raymond L, Berrino F, Benhamou E et al. (1988) Cancer of the larynx/hypopharynx, tobacco and alcohol: IARC international case-control study in Turin and Varese (Italy), Zaragoza and Navarra (Spain), Geneva (Switzerland) and Calvados (France). Int J Cancer 41: 483-491. doi:10.1002/ijc.2910410403. PubMed: 3356483. 3356483 10.1002/ijc.2910410403

[15] Rao DN, Desai PB, Ganesh B (1999) Alcohol as an additional risk factor in laryngopharyngeal cancer in Mumbai--a case-control study. Cancer Detect Prev 23: 37-44. doi:10.1046/j.1525-1500.1999.09906.x. PubMed: 9892989. 9892989 10.1046/j.1525-1500.1999.09906.x

[16] Menvielle G, Luce D, Goldberg P, Bugel I, Leclerc A (2004) Smoking, alcohol drinking and cancer risk for various sites of the larynx and hypopharynx. A case-control study in France. Eur J Cancer Prev 13: 165-172. doi:10.1097/01.cej.0000130017.93310.76. PubMed: 15167214. 15167214 10.1097/01.cej.0000130017.93310.76

[17] Boyle P, Macfarlane GJ, Maisonneuve P, Zheng T, Scully C et al. (1990) Epidemiology of mouth cancer in 1989: a review. J R Soc Med 83: 724-730. PubMed: 2250273. 2250273 10.1177/014107689008301116 PMC1292923

[18] Menvielle G, Luce D, Goldberg P, Leclerc A (2004) Smoking, alcohol drinking, occupational exposures and social inequalities in hypopharyngeal and laryngeal cancer. Int J Epidemiol 33: 799-806. doi:10.1093/ije/dyh090. PubMed: 15155704. 15155704 10.1093/ije/dyh090

[19] http://www.who.int/tobacco/mpower/en/. Monitor tobacco use and prevention policies

